# Communicating Endometriosis Pain in France and Australia: An Interview Study

**DOI:** 10.3389/fgwh.2022.765762

**Published:** 2022-03-23

**Authors:** Susanne Ilschner, Teresa Neeman, Melissa Parker, Christine Phillips

**Affiliations:** ^1^Australian National University Medical School, College of Health and Medicine, Australian National University, Canberra, ACT, Australia; ^2^College of Science, Biology Data Science Institute, Canberra, ACT, Australia; ^3^Canberra Endometriosis Centre, The Canberra Hospital, Canberra, ACT, Australia

**Keywords:** endometriosis, pain, communicating, clinical practice, diagnosis

## Abstract

Endometriosis is characterized by persistent, fluctuating pain associated with menstruation, a biological function which is socially invisible. The degree and quality of pain cannot easily be measured, observed, or documented. Difficulties in communicating pain pose particular challenges when seeking diagnosis and support from health professionals. In this paper we explore the experiences and characterization of pain by thirteen Australian and thirteen French women with endometriosis. Data were collected through semi-structured interviews using a life-history approach to illness symptoms, diagnosis and treatment. We explore the experiences of women with endometriosis in two phases: from onset of symptoms to seeking advice from a clinician, and from first consulting a clinician to receiving a diagnosis. On average, initial pain symptoms were identified 2.1 years before consulting a health practitioner, after which women reported pain symptoms 8.5 years prior to diagnosis; that is, the time between consulting a clinician and receiving a diagnosis was almost four times the period between experiencing symptoms and consulting a doctor. Pain was often “made real” to doctors by findings consistent with endometriosis on ultrasound and MRI, mostly used in France, or laparoscopy, the predominant diagnostic tool in Australia. No woman described her practitioner using standardized pain assessment tools. Thus, the validation of pain relies largely on disease visibility and the clinician-classified degree of severity rather than self-reported grades of pain or impact on activities of daily living. The invisible and enigmatic pain of this chronic women's disease remains difficult to communicate to doctors, and the recognition of severe pain is often key to timely diagnostic procedures. Clinicians need to be more proactive about severe pain related to menstruation, taking into consideration women's individual circumstances, and maintain a high index of suspicion of underlying endometriosis as a condition characterized primarily by pain.

## Introduction

Pain is often the reason to consult a medical professional. Yet pain is subjective by definition, and the degree and nature of pain are difficult to validate in a clinical setting (1, 2). In endometriosis (3–5), decisions about treatment are difficult because no curative treatment has been developed yet. Nevertheless, it is crucial for the quality of life of an individual with endometriosis that clinicians address the different forms, timing and impacts of endometriosis-related pain (6). This entails respect for a woman's views and circumstances and an understanding of the physiology, meaning and impact of pain.

That clinicians perceive and act on women's pain differently from men's pain (7, 8), and that women perceive (9, 10) and report pain differently from men is well-described. Women's pain experience may be modulated by the menstrual cycle itself (11), and reflect hormonal and metabolic differences (12), as well as ways of communicating pain (13). Chronic pelvic pain for both men and women is private, complex, and hard to communicate (14), but gender frames impacts on career paths, family roles and health care experiences differently.

Endometriosis is associated with multiple pain processes, involving inflammatory factors, ingrowth of pain fibers, and mechanical irritation by adhesions (3, 15–19). Some persistent endometriosis pain may reflect central sensitization (17, 20, 21), and may be principally reversible (22). Hence, central sensitization needs to be treated differently from other sources of pain (23).

These pain processes are fundamentally temporal: they can vary across the day, through menstrual cycles and across life stages. The priorities and coping practices of girls or women also change over time. Endometriosis has a large inheritable component (24, 25), a family history of individuals can often be found and can influence clinical decision-making for women with symptoms of endometriosis. In particular, the family narrative about menstrual pain can be influential in driving or impeding a search for diagnosis of endometriosis (26, 27).

As endometriosis is largely characterized by pain, its diagnosis relies upon clinicians recognizing and responding to descriptions of this pain (28). Pain can be reported in terms of its somatic attributes (29) or its functional impacts (30). A woman may communicate the significance of the problem by referencing the way it influences her life, and different desired outcomes. In sharing her experience of pain with a clinician, a woman may expect a diagnosis, a prognosis, a medication or a treatment plan, an exploration of the impact of comorbidities or family circumstances, or words of encouragement and compassion.

There is an emerging literature on the experiences of women with endometriosis, which highlights the breadth of its experiences upon their lives (31–33). However, there is relatively little work exploring the ways women experience their interactions with clinicians when communicating about their pain (34–38). In particular there are few comparative qualitative studies which address the experience of women with endometriosis navigating the health systems of different countries (39–41) or, more generally, the possibility of learning from patients (42). By describing patients' strategies and focus on communicating aspects of pain as important indicators of pathology we demonstrate new ways to better understand endometriosis.

Here we report the experiences of endometriosis pain described by affected women, and how this relates to communications with clinicians in Australia and France, two countries with state-supported health insurance systems, with differences in gatekeeper functions, avenues and networks of communication, and publicly-funded services. This approach locates the individuals' experience in different sociopolitical settings and throws light on the ways the health system itself and the social structures and processes may impact upon women's communication of pain.

## Methods

### Study Design

A qualitative, interview study involving semi-structured interviews of participants from France and Australia. Inclusion/exclusion criteria were: Age 18 or above with a diagnosis of, and symptoms consistent with, endometriosis. Participants needed to be able to speak English. We used the criteria and approaches recommended by Lincoln and Guba (43) to ensure the trustworthiness of our data. We undertook negative case analysis (assessment of credibility), inquiry audit by a researcher who had not undertaken the original analysis (assessment of dependability), and triangulation of methods and reflexivity (assessment of confirmability).

### Participants

Women aged 18–65 years with a diagnosis of endometriosis, confirmed by laparoscopy or imaging.

### Context

The French health system (44, 45) is based on social insurance, financed through contributions from employers and employees and applied to every French resident; residents can supplement their insurance with private contributions. Patients register with a *médicin traitant* (attending doctor, a general practitioner) whose referral to another specialist will result in reimbursement of much of the costs of the specialist consultation. No referral is required to see a gynecologist. The Australian health system is underpinned by a universal health insurance scheme, providing subsidized access to doctors. General practitioners are the main gatekeepers to specialized health care, and a referral from them is required for a consultation with specialists, including gynecologists.

### Sample Size

The sample size was determined through applying the considerations of information power for qualitative interview studies (46). Our sample had strong sample specificity, aimed for elucidation of concepts the participants already had a view on, had a high level of interview quality as defined through the nature of the interviews, and the fact that there was a single interviewer, a straightforward analysis, and was not focused on theory-generation. Under these circumstances, as sample size of 13 in each country was considered sufficient.

### Recruitment

Participants in Australia were informed about the study via a post on the endometriosis-related open Facebook group “Life with Endometriosis.” Participants in France were recruited via an open invitation to the study through group email messages distributed by EndoFrance (*Association Française de lutte contre l'Endometriose*), the French organization providing peer support and specialist advice for women with endometriosis (https://www.endofrance.org/). Participants were offered a grocery voucher as an acknowledgment.

### Data Collection

Data were collected through semi-structured remote or in-person interviews of ~45–120 min, during which participants constructed a visual timeline while reflecting on their personal biography of illness, including onset of symptoms, responses to symptoms, experiences with seeking medical help, and experience and management of pain. Interviews were conducted in person or online, in English, and were audio-recorded and transcribed after de-identification.

### Analysis

Thematic analysis was undertaken by familiarization through deep reading of the transcripts, then preliminary coding using emergent coding for each transcript. The codes were then analyzed across transcripts. Transcripts were then read again explicitly seeking outlier data which were examined to throw extra light on the themes. The initial themes arising from the analysis were: the four milestones in the early history of leave out illness (menarche, onset of symptoms, first contact with medical professional, and diagnosis), experience of pains, and accounts of contact with clinicians. The four milestones were represented visually by charting them against the age of the person at the milestone. Figures were generated using R code (47, 48). When cross-analyzed with the thematic material on contact with clinicians we established a higher order theme, *journey to diagnosis* with two components: *self-recognition of the seriousness of symptoms*, and the *clinician response prior to diagnosis*. The other thematic element, experience of pain, was analyzed by extracting the words for pain used by participants, and classifying them using the typology of pain descriptors—evaluative, sensory and affective—highlighted in the McGill Pain Questionnaire (49). Steps that were taken to increase the trustworthiness of the data collection and analysis.

### Ethical Approval

This study was approved by the Australian National University Human Research Ethics Committee (2019/933).

## Results

There were 26 participants, 13 from Australia and 13 from France. Ages for the Australian participants range from 24 to 63 Years (Median, 32), and for the French Participants From 19 to 42 Years (Median, 29) (Table 1). There Were one Aboriginal Woman and twelve Anglo-European Women in the Australian Sample, and one Woman of Caribbean Descent and twelve French-European Women in the French Sample. Sixteen Participants (6 Australian, 11 French) Had Postgraduate Education. All French Participants Were Currently Employed in the Paid Workforce or Studying Full-Time. Some Australian Participants Were Family Carers or Students While Most Were Employed in the Paid Workforce.

**Table 1:** Participants in the study.

**Participant number**	**Age group at interview**	**Age at diagnosis**	**Country of origin**
1	40–44	29	Australia
2	25–29	27	Australia
3	25–29	22	Australia
4	30–34	24	Australia
5	25–29	20	Australia
6	35–39	22	Australia
7	35–39	20	Australia
8	30–34	20	Australia
9	20–24	22	Australia
10	25–29	17	Australia
11	60–64	59	Australia
12	20–24	23	Australia
13	35–39	18	Australia
14	30–34	27	France
15	20–24	22	France
16	40–44	41	France
17	25–29	16	France
18	25–29	19	France
19	40–44	39	France
20	15–19	15	France
21	25–29	24	France
22	25–29		France
23	30–34	31	France
24	25–29	27	France
25	20–24	20	France
26	30–34	23	France

Seven Australian and three French participants had children. One woman had entered menopause, while the rest were in the reproductive period. All except the postmenopausal participant were taking hormonal medications to manage endometriosis.

**Figure 1:**
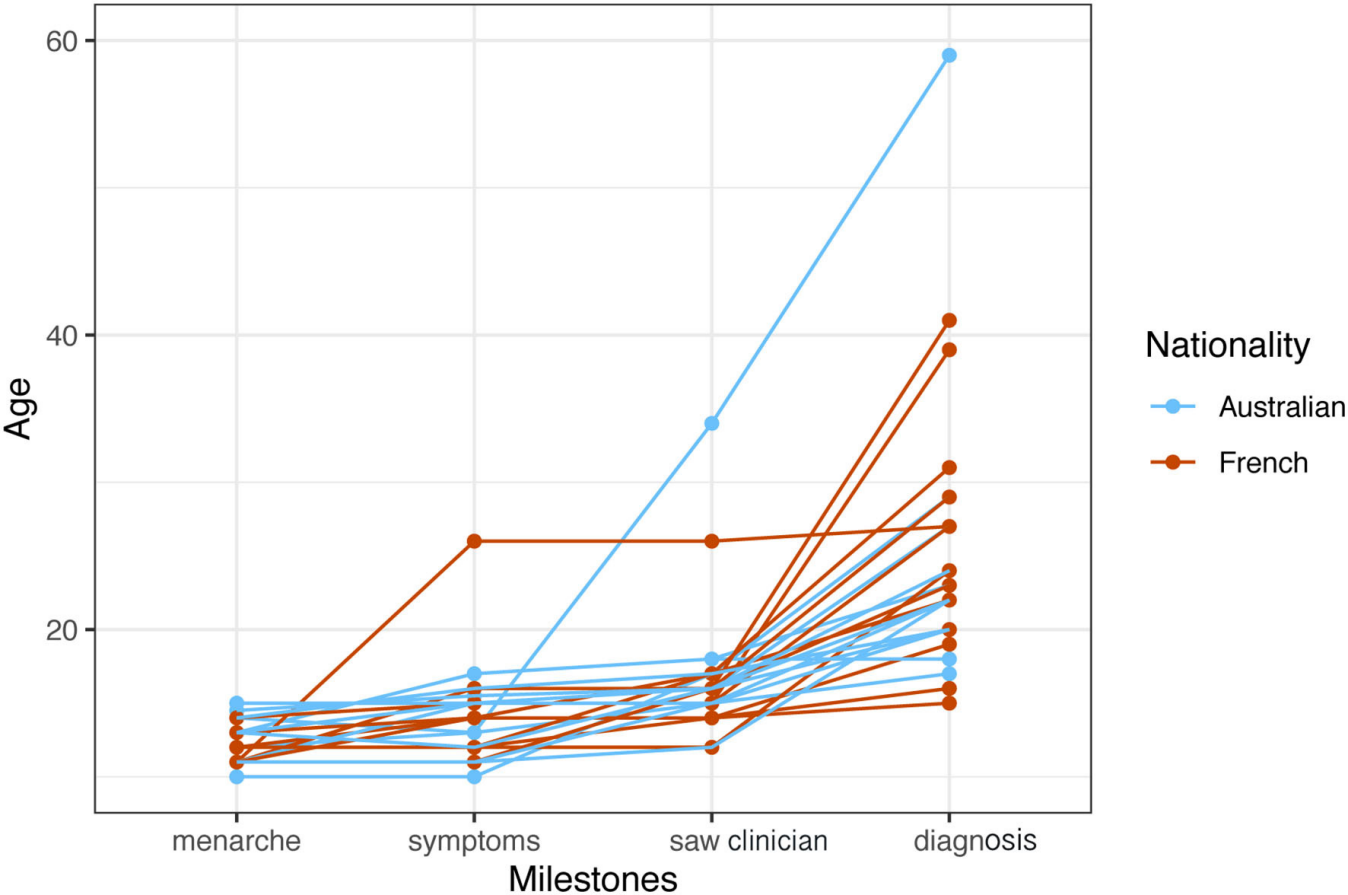
Individual trajectories of French and Australian women through life-phases to diagnosis.

Menarche occurred for participants overall at age 12.3 years (SD 1.3). Figure 1 compares the trajectory of French and Australian women through life-phases to diagnosis. Most women developed symptoms at or shortly after menarche in their teens and consulted doctors a mean period of 2.0 years later about their symptoms (range first menstruation to 7 years). The time from onset of symptoms to consulting a clinician was relatively short and uniform. Many women then experienced a significant time-lag from attending a clinician to diagnosis with an overall mean of 8.5 years. The longest delay-to-diagnosis period was reported by women over 40 years of age, reflecting diagnosis practices several decades earlier. Diagnosis in Australia was primarily achieved through the use of laparoscopy (10/13); in France the diagnosis was primarily made through MRI (9/13).

### Self-Recognition of Pain, Entering the Health System

Symptoms of endometriosis pain began early for most women, around menarche. Most women reported pain and/or heavy flow as the initial symptoms, with one woman reporting violent mood swings as her only symptom. In some cases, a contraceptive pill was taken early, and symptoms became very noticeable only after stopping the pill.

Although one woman described pain starting with menarche as being “hit by a freight train,” for most women, pain arose as a increasing period pain, which gradually extended to ovulation pain, pain during sexual intercourse and back pain.

At the beginning it was just the first day of menstruation, and then it was first day of menstruation and 1 day of ovulation, then it was three days of menstruation and so on, until I had pain every day (#26).

Some women described the severity of their pain and menstrual loss as being discounted by family.

I've been about age 11 when things all started. It started quite suddenly. So, it was extremely painful, extremely heavy. It was horrible. But whenever I complained to my parents, mostly I was told that that was normal. And I just kind of needed to buck up really (#6).

(I was told that) period pain was just a way of life and that I needed to get on with it. (*Did your family help?)* Not really, because my sister, she used to get very bad period pain as well. She doesn't have endo (#9).

You are taught everything about being a girl is painful, like your periods are supposed to be painful. The first time you have sex is supposed to be painful. So, everything is geared up that girls are supposed to experience pain (#6).

Many women realized their symptoms may be pathological when comparing their experiences with their friends' menstrual experiences.

Once one of my very close girlfriends got her period while we were out at a performance and she was like: oh my God, I need a tampon. Has anyone got anything? And I was always stocked up because my period would just come randomly, and it would always be super heavy. So, I gave her a super tampon, and she was like, What the hell is this? I can't use this. I've never used anything this big in my life. And I was like, … how can you not know what a super tampon is? … Okay, there's obviously something really wrong with me (#4).

Most women described a sentinel pain event which brought them to medical attention, often as an emergency.

When I was 19, I was hospitalized with severe abdominal pain. And I saw a general surgeon who took out my perfectly fine appendix. And he said to me, there's something going on there. I don't understand what it is, but you should see somebody (#6).

When I was 16 my mother was obliged to go to the hospital for me because of pain (#16).

### From First Consulting a Clinician to Diagnosis

In the long period to reaching a diagnosis, many women described a prolonged period when they received symptomatic treatment for menorrhagia or pelvic pain, including hormonal contraceptive treatment, or interval analgesics.

This method sometimes enabled the woman to achieve a tolerable quality of life, so there was less drive toward a diagnosis. Participants referred to “masking” the pain by painkillers and contraceptives. In addition, repeated ultrasound investigations or even MRIs which were reported as having no abnormal findings may have prevented further investigation of symptomatic women.

Several women had to weigh up pursuing a diagnosis against embarking upon a pregnancy which would have required stopping most treatments anyway. Endometriosis symptoms presenting as digestive problems sometimes led diagnostic efforts into gastroenterology, while pain-associated mental symptoms led the woman into psychiatric consultations. The costs of accessing private gynecologists, especially in Australia, were described as a factor in delaying seeking treatment.

Two system-level features of the French system supported rapid diagnosis for younger women: they could self-refer to a gynecologist and pelvic MRIs can be ordered by general practitioners. However, even when women did have radiological investigations, the diagnosis was not easy. If the tests were negative, these objective findings could operate as a way to discount symptoms, especially pain.

Just because I'd had ultrasounds and MRIs and everything, and everything always came back clear so I thought I was a hypochondriac. I was told I was a hypochondriac. I just thought it was normal, and I just had a low pain threshold. I saw many, many doctors over the years, complaining about pain (#6).

In the few cases where a diagnosis was delivered shortly after first contact with a clinician, the following conditions applied: a key person (usually a family member) had appreciated the severity of the symptoms, there was a responsive clinician, and a diagnostic test was rapidly accessed and was able to demonstrate endometriosis. In the following account, a French participant describes her diagnosis 2 years after the pain first appeared at menarche.

I knew it was not supposed to hurt that much…. My mother told me it wasn't supposed to hurt like that. That helped me a lot. … She had heard about this disease just a month ago (on the radio), and she talked about this to my gynecologist, and she told her, yes it was maybe the reason that it hurt so much. And I did an exam, it went very quick. It was like 3 months, maybe a little more. It was very quick to get a diagnosis. Actually, he is the one that helped mum birthing me. So, he knows me since I was born. He made the diagnosis by an ultrasound (in the same practice). The doctor there said it was endometriosis. Deep infiltrating. And they told the gynecologist straight away (#20).

### Communicating Pain

Table 2 presents the words used by women to describe characteristics of pain. The largest category was evaluative words, most of which described these extremes, also supported by metaphors for extremes of pain (giving birth, drinking acid or being on fire). Notable were the detailed descriptions of timing and symptom changes, including monitoring of pain levels and localization. The everyday life of someone with endometriosis and the effort to be taken seriously were frequently described using war-like metaphors; these were often coupled with terms about the recurrent nature of the pain.

**Table 2:** Ways of describing endometriosis pain.

Sensory	Electrifying, stabbing, deep, shallow, layered, pain landscape, nauseating, dizzying
Evaluative	*Negative:* Horrible, hideous, severe, immense, huge, next level, strong, massive, intense, extreme, unbearable, horrendous, terrible, awful, epic, unbelievable, incredible, agony *Neutral:* Alright, normal, latent, a bit, significant, weird, surprising, extra
Affective	Debilitating, paralyzing, filled life, became a way of life, made me hold by breath, couldn't stop it, couldn't deal with it, stressful, tiring, embarrassing, foggy, fatigued, tired, sleep-disturbed, sad, apprehensive
Location	Pelvis, (lower) back, foot, leg, belly, whole body, stomach, bladder, from adhesions inside abdomen, the bowel, under the ribs, radiating into the shoulder, all over
Fluctuation	Skyrocketed, gradually increased, always there, returned, peaked, subsided, became progressively worse, monthly, daily, with sex, with periods, flare up at any time
Metaphors	*Sensation:* Lead bar, fire, labour, drinking acid, giving birth every day *Living with endometriosis:* war, struggle, fight, battle, bullet, (pain) strikes, soldier on, revenge, race

The intensity of pain was marked by altered time perception. “It felt like a really long time, but it was probably more like an hour” (#11). Participants described motoric responses to pain. “I would be on all fours during the pain rocking back and forth” (#4). Others quantified pain in terms of type and amount of analgesia required to undertake activities of normal life. Several participants were already quite certain that they had endometriosis before seeing a specialist and one participant mentioned that she prepared beforehand to ensure that she was able to guide the specialist to recognizing that her symptoms were consistent with endometriosis.

Pain experiences which are highly intense but have a range of sensory representations proved difficult to convey to clinicians. Some women kept journals of their pain symptoms, but this was not taken into consideration in clinical consultations.

I was so concerned about developing a tolerance or a dependency to the opioids that I carried a pain chart with me on my phone. And every time I took a medication, I would put in the location of the pain, I'd rate it out of 10. And then I'd come back in an hour's time and say what the relief I got from that was, so I kind of treated myself as if I was a patient in the hospital just to demonstrate that I was utilizing the drugs in the right way (#11).

Another woman went explicitly to her GP as pain escalated using objective language (quality of life, number of painful days) and its subjective impact upon her to ask for more support.

I went to see my GP and I said to her: I need support, because I can't cope with the pain anymore…. I said to him (gynecologist): It was starting to compromise my quality of life because I've got more painful days, and it does now start to make me sad and I am more apprehensive as well #19.

No woman described a clinician using a pain inventory in consultation with her about her symptoms. Clinicians tended to pay more attention to other gynecological symptoms like heavy flow or rectal bleeding. Prior to being diagnosed, many women whose symptoms predominantly related to pain reported a deflation of their symptoms by clinicians, sometimes based on negative tests.

… they told me I was just a silly little girl who couldn't deal with pain. …fighting, for people, firstly doctors, to believe me (#6).

I kept hearing either it's normal or it's all in your head (#8).

I didn't stay that long with him (clinician) because—he was competent for sure—but he had no empathy, and he kept me for 4 months not being able to stand up (due to pain) (#21).

And I was told that no, that it (concern about endometriosis pain while on a plane trip) is just: you are terrified by the plane (#22).

He described me like that (as a limpet), and sort of basically berated me for being like that (#11).

Clinicians who were considered helpful by people with endometriosis often worked with them in addressing the impacts of endometriosis on their lives and made themselves available in case of new problems arising.

(The clinician said) Ok, you don't want children right now. All you want is not suffering. So it's our goal, and the treatment we're trying to accomplish what will fit you right now (#15).

…if you have any problem, just come back at any moment, it's not an issue. They are here for you (#14).

## Discussion

Our study focuses on the experiences and accounts of pain reported by women in Australia and France, and their journeys to diagnosis. Most women reported consulting a clinician within a few years of the onset of symptoms. Most then reported a significant delay between first consulting a clinician and a diagnosis. Our study provides evidence for the critical importance of clinicians paying attention to women's reports of pelvic pain and menstrual symptoms. It also found that the French health system, which offers simpler procedures for accessing specialist expertise and diagnostic technologies, can result in more rapid attention than the Australian health system.

This study's limitations include the fact that the women were all educated and able to participate in a long interview over the internet in English, all indicating the socio-economic status of the sample. Because the ages of our sample varied, it is important to note that some of these problems are a historical experience of older participants. They were more likely to report a longer time-to-diagnosis, perhaps reflecting lower levels of clinician knowledge about endometriosis and less developed investigative tools, as well as the contemporary move toward more active health care consumers.

Most participants described endometriosis-related symptoms and events beginning in adolescence, which spanned the entire fertile part of women's lives. Participants described predominant menstrual pain that often progressively occupied more days during the period with time and more aspects of pain accumulated over the years; thus they described a pattern at odds with normal menstrual pain (50, 51).

The first suspicion of the diagnosis of endometriosis often came from parents. Women who had family members who normalized severe menstrual pain were more likely to realize that their experience might be abnormal by comparing with friends, through information from social contacts or the media. In a 2013 Australian study, young women often had vague or inaccurate concepts of endometriosis and expressed a desire to have readily accessible information available to access online (52). A growing movement of peer-to-peer education through social media is driving access to knowledge and social support for endometriosis in ways that have not previously been available to women (53, 54).

The reasons underpinning delays in diagnosis after women had presented with symptoms to the health system are polyfactorial. Participants described many lost opportunities. Reports of crises and hospitalizations due to severe pain underscore the urgency of seeking the cause of pain. Despite the extra attention and effort typically not much was achieved by Emergency Department attendances, and opportunities to communicate the extent of the problems were lost.

The mean diagnostic delay in our study is consistent with that reported elsewhere (55) for women with surgically-confirmed endometriosis. Given the possibility of easier access but more diagnostic error with radiological investigations (56), there is an urgent need for communication of the significance and limitations of the contemporary imaging techniques and risks of laparoscopy. Patients are mostly excluded from this discussion during consultations, although they will have to weigh up risks and options for themselves. Participants in our study did report awareness of this issue.

Although there were substantial delays in both countries in obtaining a diagnosis, the French health system is more conducive to earlier diagnoses of endometriosis. The national French health system enables self-referral to a gynecologist and does not place restrictions around the ordering of MRIs by general practitioners. In Australia, a definitive diagnosis can only be achieved by a gynecologist (on referral from a general practitioner), with an apparent preference for laparoscopic diagnosis. This means for a patient that she has to overcome several obstacles to convince clinicians of her invisible symptoms. Women prepare for this struggle by carefully assembling evidence, arguments and ways to elicit empathy.

Thus, in Australia women face multiple steps through a health system to reach diagnosis, requiring a coalition of clinicians to agree that endometriosis is a diagnostic possibility, whereas French women face only one or two steps. Earlier diagnosis in Australia would occur if the Australian health insurance system subsidized referrals for specialist ultrasound and MRI examinations from general practitioners to investigate endometriosis, and if specialized endometriosis services were more readily available to Australian women through self-referral. For women with severe, long-term endometriosis requiring costly treatment the French health system also affords extra support through recognition of their condition as an *Affection Longue Durée*.

Several women in our study had developed their own monitoring systems for pain. Tools such as the McGill Pain Inventory are reliable instruments for describing pain; indeed, women in this study spontaneously demonstrated competence in using terminology across the three domains of this inventory, and beyond. Their use of war-like metaphors is in line with those frequently used in medical literature (57, 58) to address difficult, significant problems, including communication with clinicians (59, 60). This is not, then, an example of incommensurable medical discourses between a patient-centered discourse on pain and a clinician-centered discourse on signs (61). Instead, it appears to be a failure of imagination on the part of the clinicians consulted by our informants who often explained away symptoms on the basis of existing negative tests or interpretations of pain behaviors. In a French study preoperative endometriosis patients gave largely overlapping but more complete descriptions of their pain symptoms than expert gynecological surgeons (62). Content reviews of medical curricula in a number of countries indicate relatively little attention to menstrual health (63, 64). Medical curricula are known to under-address pain management and responses to pain in clinical skills training (65, 66). This combination of under-representations creates a risk for women with endometriosis symptoms characterized by pain, with graduates having little preparation for responding to the needs of their patients with endometriosis symptoms.

We urge clinicians to collaborate with their patients with severe menstrual symptoms in monitoring their pain using standardized tools as well as patient developed indicators and assessing with them its impacts upon their quality of life. Details of individual circumstances and life stage are also important to guide the clinician's attempt help their patients. Endometriosis has complex impacts on the lives of many women. Attention to and respect for the reported experiences of women can assist in containing and handling the pain associated with it and drive earlier diagnosis.

## Data Availability Statement

The datasets presented in this article are not readily available because data might be identifiable. Requests to access the datasets should be directed to SI, susanne.ilschner@anu.edu.au.

## Ethics Statement

The studies involving human participants were reviewed and approved by Australian National University Human Research Ethics Committee (2019/933). The patients/participants provided their written informed consent to participate in this study. Written informed consent was obtained from the individual(s) for the publication of any potentially identifiable images or data included in this article.

## Author Contributions

SI conceived the study and conducted the interviews. SI, MP, CP, and TN analyzed the interview data. SI and CP wrote the article. All authors contributed to the article and approved the submitted version.

## Funding

SI was funded through an Australian Government Research Training Program (AGRTP) Stipend Scholarship.

## Conflict of Interest

The authors declare that the research was conducted in the absence of any commercial or financial relationships that could be construed as a potential conflict of interest.

## Publisher's Note

All claims expressed in this article are solely those of the authors and do not necessarily represent those of their affiliated organizations, or those of the publisher, the editors and the reviewers. Any product that may be evaluated in this article, or claim that may be made by its manufacturer, is not guaranteed or endorsed by the publisher.
